# Integrated Genome and Transcriptome Sequencing to Solve a Neuromuscular Puzzle: Miyoshi Muscular Dystrophy and Early Onset Primary Dystonia in Siblings of the Same Family

**DOI:** 10.3389/fgene.2021.672906

**Published:** 2021-07-02

**Authors:** Feng Zhu, Fengxiao Zhang, Lizhi Hu, Haowen Liu, Yahua Li

**Affiliations:** ^1^Department of Cardiology, Union Hospital, Tongji Medical College, Huazhong University of Science and Technology, Wuhan, China; ^2^Clinic Center of Human Gene Research, Union Hospital, Tongji Medical College, Huazhong University of Science and Technology, Wuhan, China; ^3^Department of Neurology, The Third Hospital of Hebei Medical University, Shijiazhuang, China; ^4^Department of Respiratory Medicine, The Third Hospital of Hebei Medical University, Shijiazhuang, China

**Keywords:** Miyoshi muscular dystrophy, dysferlin, deep intron mutation, compound heterozygous mutations, dystonia

## Abstract

**Background:**

Neuromuscular disorders (NMD), many of which are hereditary, affect muscular function. Due to advances in high-throughput sequencing technologies, the diagnosis of hereditary NMDs has dramatically improved in recent years.

**Methods and Results:**

In this study, we report an family with two siblings exhibiting two different NMD, Miyoshi muscular dystrophy (MMD) and early onset primary dystonia (EOPD). Whole exome sequencing (WES) identified a novel monoallelic frameshift deletion mutation (*dysferlin*: c.4404delC/p.I1469Sfs^∗^17) in the Dysferlin gene in the index patient who suffered from MMD. This deletion was inherited from his unaffected father and was carried by his younger sister with EOPD. However, immunostaining staining revealed an absence of dysferlin expression in the proband’s muscle tissue and thus suggested the presence of the second underlying mutant allele in dysferlin. Using integrated RNA sequencing (RNA-seq) and whole genome sequencing (WGS) of muscle tissue, a novel deep intronic mutation in *dysferlin* (*dysferlin*: c.5341-415A > G) was discovered in the index patient. This mutation caused aberrant mRNA splicing and inclusion of an additional pseudoexon (PE) which we termed PE48.1. This PE was inherited from his unaffected mother. PE48.1 inclusion altered the Dysferlin sequence, causing premature termination of translation.

**Conclusion:**

Using integrated genome and transcriptome sequencing, we discovered hereditary MMD and EOPD affecting two siblings of same family. Our results added further weight to the combined use of RNA-seq and WGS as an important method for detection of deep intronic gene mutations, and suggest that integrated sequencing assays are an effective strategy for the diagnosis of hereditary NMDs.

## Introduction

Neuromuscular disorders (NMD) are a general term for a group of conditions that result in impaired muscle function, either directly due to pathology of voluntary muscles or indirectly due to pathology of the peripheral nervous system or neuromuscular connections. Several NMDs are caused by hereditary mutations and result in progressive muscle degeneration and weakness.

Early onset primary dystonia (EOPD) is an autosomal dominant genetic NMD caused by a number of mutations (Bressman et al., 2009). It usually manifests with weakness in the legs or arms and over time progresses to the trunk and other limbs. Systemic dystonia can disable sufferers and limit their mobility and independence. The first dystonia gene, *TOR1A* (also known as *DYT1* or *DQ2*), which causes systemic dystonia with average age of onset at 13, was discovered in 1997 (Ozelius et al., 1997; Cheng et al., 2014). Due to a lack of specific imaging or laboratory manifestations of EOPD, clinical diagnosis cannot be made via by imaging or routine laboratory tests and genetic testing is thus critical for diagnosis.

Miyoshi muscular dystrophy (MMD) is an autosomal recessive genetic NMD caused by mutation of the *dysferlin* gene located on chromosome 2 (Bashir et al., 1998). *dysferlin* encodes the Dysferlin protein which is located in the membrane and cytoplasmic vesicles of muscle cells and is involved in membrane fusion and repair. The clinical manifestations of the MMD are diverse, with several types of electromyogram (EMG) potentially highlighting myogenic lesions. As the clinical symptoms of dystrophy are not unique, skeletal muscle pathology, molecular pathology, molecular genetics and other diagnostic techniques are required for clinical diagnosis of MMD.

Traditional gene mapping via construction of genetic linkage maps is complicated, time-consuming and labor-intensive, and in many cases inadequate for clinical diagnosis as disease modulating variants for many diseases are still largely unknown. Whole exome sequencing (WES) represents a significant technical advance that has greatly improved genetic diagnostics. Nonetheless, there remains a large group of patients for whom underlying genetic causes of their diseases have not been uncovered despite through investigation with either of these modalities. One potential and underexplored source of mutations are variants which alter RNA expression and/or processing. Such mutations may occur at exon/intron boundaries (and thus be captured by WES), or occur outside of the standard exome, such as in deep intronic and intergenic regions, and would not be captured using WES. Whole genome sequencing (WGS) offers advantages over WES for detection of such intronic or intergenic mutations, but only few genetic disorders are caused by intron sequence mutations and WGS is thus not always first choice for genetic analyses. Approximately 15% of mutations listed in the Human Gene Mutation Database are described at splice-site junctions (Gonorazky et al., 2016). RNA sequencing (RNA-seq) has become an essential tool for the assessment of differential gene expression and mRNA splicing at the whole transcriptome level (Oshlack et al., 2010; Kukurba and Montgomery, 2015). It helps to understand the full range of RNA biology, including transcriptional alterations, splice variants, and molecular interactions. Current state-of-art of sequencing technologies such as WES, WGS, and RNA-seq, can thus elucidate the whole spectrum of variants in a given individual which may stimulate the discovery of novel genetic causes.

Here, we report on the analysis of mutation profiles using high-throughput genome and transcriptome sequencing in a family with two siblings with two different NMD, EOPD and MMD. Our study emphasizes that integrated sequencing assays are an effective modality for the genetic diagnosis of clinical rare genetic diseases.

## Subjects and Methods

### Clinical Evaluations

**FIGURE 1:**
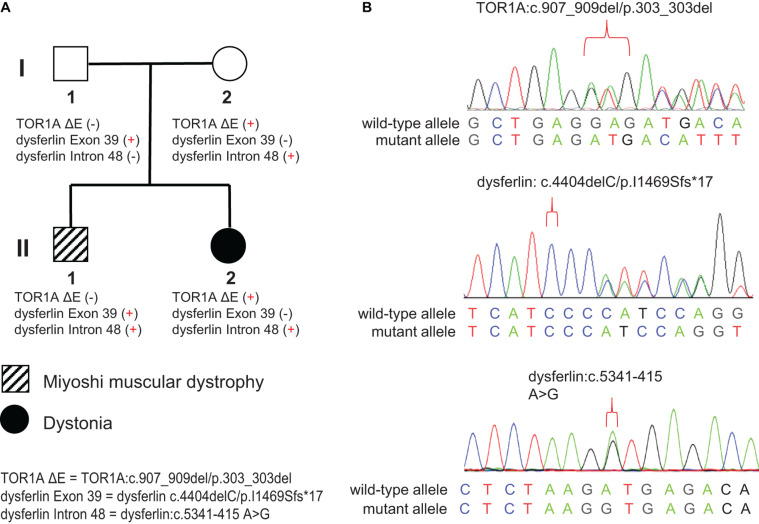
Pedigree and Sanger sequencing chromatograms of the identified disease-causing variants. **(A)** Two different disease phenotypes were found within the same family, namely Miyoshi muscular dystrophy, which affected II-1, and dystonia, which affected II-2. II-1 harbored compound heterozygous variants in the *DYSF* gene (c.4404delC/p.I1469Sfs*17 and c.5341-415 A > G), derived separately from his parents. II-2 harbored the c.907_909del/p.303_303del variant in the *TOR1A* gene and the c.4404delC/p.I1469Sfs*17 variant in the *DYSF* gene, both of which were inherited from her mother. **(B)** Sanger sequencing chromatograms of the c.907_909del/p.303_303del variant in the *TOR1A* and the c.4404delC/p.I1469Sfs*17 and c.5341-415 A > G variants in the *DYSF* gene.

The Ethical Committee of the Tongji Hospital, Tongji Medical College, Huazhong University of Science and Technology, Wuhan, China, reviewed and approved our study protocol. All participants provided written informed consent. The family was identified after the evaluation of motor symptoms in one male index patient (II-1, Figure 1). All available family members (I-2, II-1, and II-2) underwent a neurological examination, EMG, magnetic resonance imaging (MRI) for skeletal muscle and brain, and donated a blood sample for further molecular analysis.

### High-Throughput Sequencing

For WES and WGS, genomic DNA was prepared from peripheral blood samples by using TIANamp Blood DNA Kit (Tiangen, China). WES was performed for identification of disease-causing mutations in the protein-coding regions of the genome. Genomic DNA from each individual was used for the initial enzymatic digestion and the whole exome was captured using the xGen^®^ Exome Research Panel v1.0 (IDT, United States). The quality and quantity of WES libraries was assessed on the Agilent 2100 bioanalyzer (Agilent, United States). WES was performed on the NovaSeq 6000 platform (Illumina, United States) using 150-bp paired-end reads. WGS was carried out for identification of structural or non-coding variants related to disease phenotype. Extracted genomic DNA from peripheral blood samples was sheared to the target size (250 bp) using the Q800R sonicator (Covaris, United States) and the size of sheared genomic DNA fragments was assessed with a Agilent 2100 bioanalyzer (Agilent, United States). WGS libraries were constructed and sequencing was performed on the NovaSeq 6000 platform (Illumina, United States) using 75-bp single-end reads. To detect the alternative RNA splicing variants associated with disease phenotype, RNA sequencing was performed on muscle biopsy samples. Total RNA was extracted with Trizol (Invitrogen, United States). Quantity and quality of the extracted RNA was assessed with the Agilent 2100 bioanalyzer (Agilent, United States). Poly(A) mRNAs was enriched using oligo-dT magnetic beads (Invitrogen, United States) according to the manufacturer’s instructions and cDNA libraries were then prepared using the TruSeq Stranded mRNA library kit (Illumina, United States). Sequencing was performed on the Illumina NovaSeq6000 (Illumina, United States) using 150-bp paired-end reads.

### Bioinformatics Data Analysis

To identify disease mutations, sequencing data was analyzed and annotated using an in-house pipeline. Briefly, reads of WES and WGS were mapped to the GRCh37/hg19 reference sequences using the Burrows-Wheeler Aligner (Pertea et al., 2016). For analysis of WES data, the SAM tools pipeline as well as ANNOVAR and several prediction tools were used to call single nucleotide variants (SNVs) and indels (<50 bp) (Shigemizu et al., 2013; Yang and Wang, 2015). Notably, each variant was compared against two public databases, the 1,000 genomes project^1^ and Exome Aggregation Consortium^2^. Based on the variant annotations, a series of filtering strategies were applied to screen candidate variants associated with the disease phenotype, as described previously (Requena et al., 2017). Variants which fulfilled the following criteria were considered for further analysis: (1) variants were not outside exonic and splicing regions; (2) variants were not synonymous; (3) variants did not exhibit a minor allele frequency more than 1% based on public databases; (4) variants were not non-conservative, with score ≤2 based on to GERP++ conservation prediction; and (5) variants did not cause protein function loss as predicted by SIFT, Polyphen-2, LRT, or MutationTaster. The remaining data formed a list of candidate variants and related genes. To prioritize the most likely candidate disease-causing genes, all candidate genes were ranked using Phenolyzer (Yang et al., 2015).

For analysis of RNA-seq, raw reads were mapped against the GRCh38/hg19 human reference genome using HISAT2 (Exposito et al., 2018). Aligned reads were assembled and quantified using StringTie (Pertea et al., 2015). Alternative pre-mRNA splicing events were analyzed using Human Splicing Finder (Desmet et al., 2009) and visualized using the Integrative Genome Viewer (IGV) (Romero et al., 2016).

### Histochemical and Immunohistochemical Staining

A muscle biopsy was taken for routine diagnostic purposes in the index patient (II-1). Sections were stained using conventional histochemical and immunohistochemical methods.

For Hematoxylin and eosin staining, cryosections of muscle biopsy samples (5–10 μm) were stained with hematoxylin and eosin, as described previously (Sundaram et al., 2011). For nicotinamide-adenine dinucleotide staining, modified Gomori trichrome staining and Neuron-specific enolase staining, cryosections of the muscle biopsy sample were prepared and stained as described previously (Tsokos et al., 1984; Lee et al., 2016; Lv et al., 2020).

For immunohistochemistry, the muscle biopsy sample was fixed in 4% paraformaldehyde for 24 h and subsequently paraffin-embedded. Sections (5 μm) were treated with 0.3% hydrogen peroxide to prevent endogenous peroxidase activity and 0.01 M citrate buffer restore antigenic activity. Sections were blocked in 2% BSA and incubated with the following antibodies overnight at 4°C: anti-dystrophin-C(abcam, United States), anti-dystrophin-N(abcam, United States), anti-dystrophin-R(sigma, United States), anti-sarcoglycan-α(Santa Cruz, United States), anti-sarcoglycan-β(Santa Cruz, United States), anti-sarcoglycan-γ(Santa Cruz, United States), anti-sarcoglycan-δ(Santa Cruz, United States) and anti-Dysferlin(abcam, United States). Sections were incubated with the appropriate HRP-conjugated secondary antibodies for 30 min, followed by treatment with alkaline phosphatase substrate (DAB) for visualization.

### Reverse Transcription-Polymerase Chain Reaction (RT-PCR) and Sanger Sequencing

RNA was extracted from peripheral blood mononuclear cells from II1 and health control using a RNA Extraction Kit (Takara Bio). RNA was transcribed into cDNA by PrimeScript^TM^ RT Master Mix (Takara RR036A) and the PCR was carried out as described previously (Chen et al., 2021). Primers targeting exon 48 and exon 49 of *dysferlin* was designed as follows: forward 5n′-CTGCCTCTGGAGAAGGA-3′; reverse 5′-GTACACAGGTGCCTTGA-3′.

DNA was extracted from peripheral blood mononuclear cells from all available family members using a Genomic DNA purification kit (Takara Bio). To validate the identified variants, Sanger sequencing was performed on DNA samples as described previously (Xiong et al., 2019). The primer pairs for PCR and Sanger sequencing were as follows:

*TOR1A* (c.907_909del/p.303_303del): forward 5′-CTCC CCCTGGAATACAAACA-3′, reverse 5′-CCAGGGAG AATTCCTGTCAC-3′;
*dysferlin* (c.4404delC/p.I1469Sfs^∗^17): forward 5′-CTGC AGGGTCTTGTCTTGGT-3′, reverse 5′GAGAAGGGG TGGGAATTGAT-3′;
*dysferlin* (c.5341-415A > G): forward 5′-CCAAAGT GAGCCATGAGGAT-3′, reverse 5′-TGTCAAAGAGCAC CGTTCAG-3′.

## Results

### Clinical Findings

**FIGURE 2:**
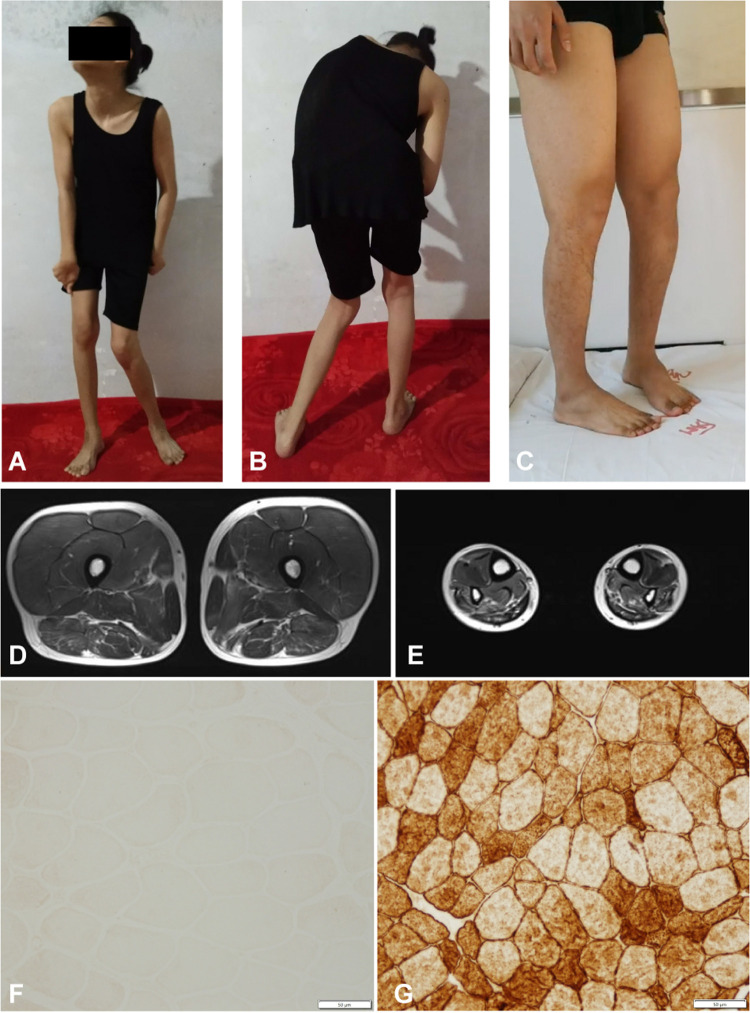
Clinical features of the familial member II-1 and II-2. **(A,B)** Photographs of the familial member II-2 showing clinical features of generalized dystonia. **(C)** Photograph of the familial member II-1 showing muscle atrophy in both distal lower limbs. **(D,E)** MRI revealed normal proximal muscle in the lower extremities and atrophy of the distal limbs in the familial member II-1. **(F)** Immunostaining of muscle biopsy of the familial member II-1 demonstrated absence of Dysferlin (Bar, 50 μm). **(G)** Immunostaining of muscle biopsy of a healthy volunteer showed normal Dysferlin staining (Bar, 50 μm).

The pedigree of the family is shown in the Figure 1A. There were two different NMD present in the studied family: the index patient (II-1) was diagnosed with MMD while his younger sister (II-2) was diagnosed with EOPD (Figure 1A). The index patient (II-1), a 25-year-old Chinese male, reported limb weakness, chest tightness, and suffocation symptoms after exercise at age 18. At examination 4 years later, the symptoms of limb weakness and chest tightness progressed and became worse, and the patient reported an inability to stand on his toes and myalgia in his legs aggravated by exercise. The examination revealed wasting of gastrocnemius and soleus muscles (Figure 2C) which was further confirmed by MRI (Figures 2D,E). Serum CK levels ranged from 8,650 to 14,684 u/l, which constituted a 50- to 85-fold increase compared to reference levels. EMG showed no changes in nerve conduction velocity and therefore neurogenic myoatrophy was ruled out. The index patient’s younger sister (II-2), a 22-year-old Chinese female, was the first in the family to be affected with an NMD. Her disease onset occurred at age 9 in right arm. At examination 12 years later, both upper limbs, the right leg, neck, and trunk were affected (Figures 2A,B). Limb dystonia was relieved with sleep. The patient’s sister also exhibited intermittent lip puckering, a tremulous voice, and involuntary toe movements and a decrease in muscle mass in the lower arms and legs was noted in the examination (Figures 2A,B). EMG indicated a large number of myotonic potentials in many parts of the body. However, serum CK levels were normal and the patient did not respond to levodopa treatment. Combined botulinum toxin type A and low-frequency repetitive transcranial magnetic stimulation also failed to improve dystonia symptoms. The index patient’s parents (I-1 and I-2), both aged 47, were unaffected by dystonia or distal muscle dystrophy, and serum CK levels of I-1 and I-2 were normal for their age and gender.

We then performed a diagnostic muscle biopsy from the left bicep in the index patient (II-1). Hematoxylin and eosin staining revealed that the size of muscle fibers varied greatly, and the diameter was 20–80 microns (Supplementary Figure 1A). We observed scattered cellular degeneration, necrosis, and regeneration in the muscle tissue (Supplementary Figures 1A–C). Staining for reduced forms of nicotinamide-adenine dinucleotide demonstrated an uneven distribution of oxidase in several muscle fibers (Supplementary Figure 1D). However, there was no inflammatory cell infiltration, no broken red-edged fibers, no fat tissue infiltration, and muscle glycogen levels was roughly normal (Supplementary Figures 1A–D). Both dystrophin and sarcoglycan immunostainings were normal (Supplementary Figures 1E,G–K). Compare to the healthy control, the dysferlin immunostaing of muscle biopsy of II-1 showed an absence of the Dysferlin protein (Figures 2F,G). Based on clinical symptoms and muscle biopsy results, the diagnosis of MMD was suspected for II-1.

### WES and Filtering for Deleterious Candidate Variants Related to the Disease Phenotype

WES was performed on both family members affected by muscular disorders, II-1 and II-2. An average of 72,786 variants were identified for each individual. As described in methods, 493 variants originating from 348 genes and 376 variants originating from 257 genes were kept for II-1 and II-2, respectively. These genes were then associated with the “neuromuscular disease” phenotype using Phenolyzer. We found a non-frameshift deletion (c.907_909del/p.303_303del) variant of the *TOR1A* gene located on chromosome 9q34(GRCh37/hg19, chr9:132576341-132576343) in II-2. This deletion, also known as deltaE303 (ΔE), or rs80358233, is in the most common pathogenic variant associated with EOPD (Yang et al., 2009). WES data from index patient II-1 revealed a novel exonic variant of the *dysferlin* gene located on chromosome 2p13(GRCh37/hg19, chr2:71840531) which introduces a premature stop codon and is considered to be related to MMD (*dysferlin*: c.4404delC/p.I1469Sfs^∗^17). This frameshift deletion was monoallelic in II-1 and was also found in WES data from II-2.

### Identification of *dysferlin* Mutations in the Index Patient

Based on immunostaining staining and WES results, we suspected that *dysferlin* was the pathogenic gene causative of MMD in the index patient II-1. As the Dysferlin protein was absent in muscle tissue of II-1, we expected a the second underlying mutant allele in *dysferlin* in addition to c.4404delC/p.I1469Sfs^∗^17 which the patient inherited his mother.

**FIGURE 3:**
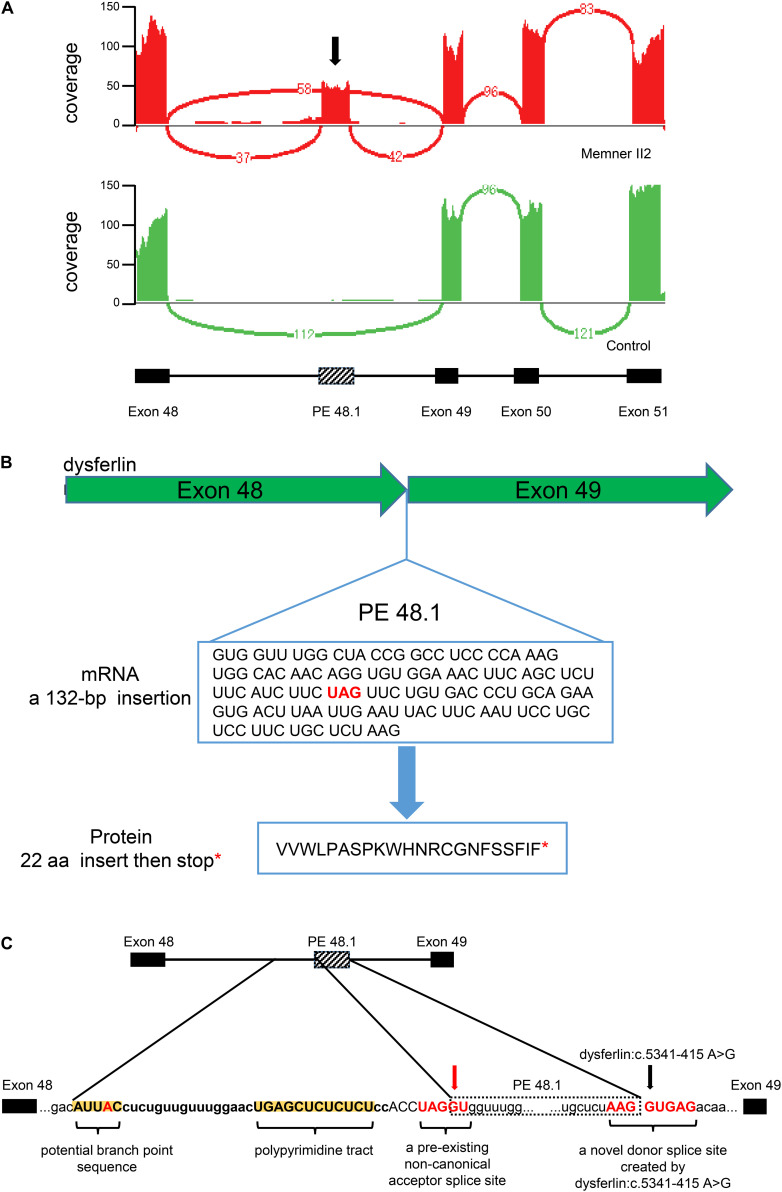
Integrated RNA-seq and WGS identify a pathogenic deep intronic variant in the *DYSF* gene in the familial member II-1. **(A)** Sashimi plot generated using IGV illustrates RNA-Seq reads mapping to the *DYSF* locus. Splice junctions are displayed as arcs connecting exons. Sashimi plots generated by IGV for two RNA-Seq samples. Splicing events of the *DYSF* gene in the familial member II-1 and the healthy control (red and green, respectively). Exon 48, 49, 50, and 51 of the *DYSF* gene are shown as RefSeq annotation tracks. A pseudoexon inclusion, which we term PE 48.1, is indicated by a black arrow in Sashimi plot (red). **(B)** RNA-seq revealed a 132 base pair insertion, PE 48.1, between exon 48 and exon 49. PE48.1 encodes 22 additional amino acids followed by a stop codon (red asterisk). **(C)** WGS, which covers the entire *DYSF* sequence, revealed a novel donor splice site created by *DYSF*: c.5341-415 A > G (black arrow). The intronic sequence upstream of PE44.1 contains additional consensus sites required for mRNA splicing, including a pre-existing non-canonical acceptor splice site (red arrow), an adjacent pyrimidine-rich region (yellow highlight) and one potential branch point sequence (yellow highlight) that could be used to promote splicing.

Transcriptomic abnormalities can be captured by RNA-seq. We therefore performed RNA-seq on II-1 and an unrelated healthy male volunteer as a control. Examination of the *dysferlin* mRNA transcripts revealed a 132-bp novel pseudoexon (PE) insertion which we termed PE 48.1. Sashimi plots generated using IGV illustrate the location of PE 48.1 between exons 48 and 49 of dysferlin mRNA transcripts (Figure 3A). Inclusion of PE48.1 in the mRNA transcript is predicted to lead to insertion of 22 additional amino acids followed by a stop codon encoded by the pseudoexon sequence (Figure 3B). We next performed WGS to identify the variant responsible for this aberrant splicing event in *dysferlin*. Among all 361 identified variants in the *dysferlin* gene (Supplementary Figure 2), a novel point variant (c.5341-415A > G, GRCh37/hg19, chr2:71895469) deep within intron 48 was predicted to be a splicing donor site variant by the Human Splicing Finder tool (Desmet et al., 2009). Along with this novel splicing donor site variant caused by an A > G transition, analysis of the intronic sequence upstream of PE 48.1 revealed the other sequence elements required for splicing in this region (Figure 3C), for example there was a pre-existing non-canonical acceptor splice site at the 5′ end of PE48.1 as well as a pyrimidine-rich region and one potential branch point consensus sequence in the upstream of PE48.1. Both these sequences, in the presence of the deep intronic variant c.5341-415A > G, allow splicing of the new pseudoexon PE48.1 between exon 48 and exon 49 (Figure 3). To further confirm the results, we examined the expression of *dysferlin* mRNA in peripheral blood mononuclear cells from II1 and health control by RT-PCR. Gel electrophoresis showed that PCR product of cDNA from II1 had two bands 2 different bands, and the upper band was the abnormal *dysferlin* (Supplementary Figure 2).

Sanger sequencing of the familial members showed that the frameshift deletion variant (*dysferlin*: c.4404delC/p.I1469Sfs^∗^17) was present in the index patient’s father but not his mother (Figures 1A,B). Meanwhile, the splicing donor site variant (*dysferlin*: c.5341-415A > G) was present in the index patient’s mother but not his father. These results confirmed that the index patient inherited two mutations—a paternal and a maternal mutation—in different copies of the *dysferlin* gene (Figures 1A,B).

Therefore, we concluded that the compound heterozygous variants of the *dysferlin* gene c.4404delC and c.5341-415A > G may lead to the observed deficient Dysferlin protein expression, consistent with immunostaining results.

## Discussion

In the present study, we analyzed genomic and transcriptomic profiles using high-throughput sequencing in a family with two different NMD, MMD and EOPD. Using integrated genomic and transcriptomic analyses, we identified two novel compound heterozygous *dysferlin* mutations in the male index patient with MMD. Dysgenic mutations and a heterozygous the ΔE mutation in the *TOR1A* gene and frameshift deletion mutation (*dysferlin1*: c.4404delC/p.I1469Sfs^∗^17) in the *dysferlin* gene were identified in the index patient’s sister with severe dystonia. The patient’s mother, who carried the ΔE mutation of the *TOR1A* gene and the splice site mutation of *dysferlin*, was asymptomatic. Likewise, the patient’s father, who carried the *dysferlin* frameshift deletion mutation, was also asymptomatic.

EOTD, also known as Oppenheim’s dystonia, is characterized by limb-onset dystonia in childhood, with subsequent progression to generalized dystonia (Kamm, 2006). A deletion of three basepairs (GAG) in the *TOR1A* gene on chromosome 9q34 has been demonstrated to be the founding mutation of EOPD and is responsible for the majority of typical EOPD cases of diverse ethnic origins (O’Riordan et al., 2002). The deletion mutation removes a single in-frame amino acid (glutamate 302 or 303, ΔE) from the carboxyl terminal region of the encoded protein, TorsinA. TorsinA belongs to the AAA+ (ATPase associated with different cellular activities) protein family. AAA+ TPases typically use energy from ATP hydrolysis to drive cellular activities, such as protein trafficking, protein folding, and protein degradation, amongst other functions (Miller and Enemark, 2016). It has been shown that expression of TorsinA containing the dystonia-causing ΔE mutation results in increased cellular deformability and susceptibility of to damage by mechanical forces (Gill et al., 2019). The clinical presentation of EOPD is remarkably variable, ranging from mild dystonia limited to a single body region, such as the neck, to widespread and severe involvement of limb, axial, and cranial muscles (Ozelius and Bressman, 2011). Despite an autosomal dominant inheritance, only 30% of individuals who carry the ΔE-TorsinA mutation will develop EOPD, even within affected families (Eidelberg et al., 1998; Ghilardi et al., 2010). This effect is known as reduced penetrance, suggesting that modifying factors (environmental and/or genetic) are responsible for penetrance as well as the onset patterns and severity of this disorder (Cooper et al., 2013). Although no of environmental causes for modification of penetrance have been defined so far, several *TOR1A* variants have been associated with penetrance. For example, a SNP in position 216 of *TOR1A* (rs1801968) causing replacement of D with H has been shown to be associated with EOPD penetrance (Kamm et al., 2008), suggesting this SNP may act as potent intragenic modifier. However, neither the index patient’s mother nor sister carried this *TOR1A* SNP.

As introns do not encode proteins, they were initially considered non-functional regions (Tilgner et al., 2012). Recent studies in transcriptome sequencing and analysis have revealed that deep intronic mutations can result in inclusions of pseudoexons, create new splice sites, and alter transcriptional regulatory motifs and the activity of non-coding RNA genes (Hsiao et al., 2016; Naro et al., 2017). Deep intronic mutations have since been documented in multiple diseases (Vaz-Drago et al., 2017) and therapeutic strategies for treatment of diseases associated with deep intronic mutations are gaining momentum. For example, for cancers caused by certain *BRCA2* mutations, specific antisense oligonucleotides (ASO) have been shown to block cryptic exon inclusion caused by deep intronic mutations *in vitro* (Anczukow et al., 2012). Moreover, work by Matos et al. (2014) demonstrated that modified U1 snRNA vectors may serve as a therapeutic tool to reconstitute the normal splicing process. Additionally, molecular chaperone assays using glucosamine for mutant proteins have been shown to result in the restoration of function of misfolded proteins (Matos et al., 2014). Lastly, viral gene transfer of full-length genes has been used to restore function of wild type proteins (e.g., dystrophin protein for treatment of Duchenne Muscular Dystrophy) (Nakamura, 2019). Thus, there are several promising therapeutic strategies for the correction of deep intronic mutations associated with pathogenic variants.

In order to discover deep intronic mutations and their pathogenicity, it is essential to integrate DNA sequencing of intronic regions and mRNA analysis derived from pathological tissue. The detection of novel deep intronic mutations may be achieved by conventional RT-PCR analysis or by RNA-seq; conventional laboratory detection methods in the past combined PCR and Sanger sequencing, which was time-consuming and labor-intensive. However, with new high-throughput sequencing technologies, deep intronic mutation sites can be identified in a relatively convenient way (Gonorazky et al., 2016), and RNA-seq has been previously used to identify deep intronic mutation sites of myopathy (Marco-Puche et al., 2019).

In this study, using integrated genome sequencing and RNA-seq, we identified a deep intronic *dysferlin* mutation (*dysferlin*: c.5341-415A > G) which results in inclusion of a pseudoexon. This mutant variant and a frameshift deletion in *dysferlin* (*dysferlin*: c.4404delC/p.I1469Sfs^∗^17) caused abnormal deficiency of Dysferlin expression in diseased tissue. Results from our study suggest that the exploration of deep intron mutations may contribute to further understanding of human genetic diseases.

Dysferlinopathies are caused by mutations in *dysferlin* and include a spectrum of muscle disease characterized by two main phenotypes, MMD and limb-girdle muscular dystrophy type 2B (LGMD2B). Dysferlinopathies are clinically heterogeneous, which is why they can be easily misdiagnosed or missed (Bashir et al., 1994). Recent studies have shown that intronic splicing alterations can that lead to muscular dystrophies. Dominov et al. (2014) identified that an intronic variant of Dysferlin (44i, c.4886 + 1249G > T) induces alternate splicing of the Dysferlin transcript, resulting in an in-frame insertion of 59 amino acids within the C2F domain of the Dysferlin protein. Dominov et al. (2019) also found a second deep intronic DYSF mutation (in intron 50) leading to expression of another pseudoexon (PE50.1).

There are also many pathogenic intronic mutations in the other muscular dystrophies. Cummings et al. (2017) identified a novel intronic mutation of *COL6A1* that results in a dominantly acting gain of splicing, contributing substantially to inherited diseases. Moreover, a homozygous deep intronic single base pair deletion in *CAPN3* (c.946-29delT) may lead to limb girdle muscular dystrophy (Hu et al., 2019). Fitzgerald et al. (2020) report a novel intronic variant in the MTM1 gene, which leads to reduction of myotubularin-1 protein, and results in the X-linked neonatal myopathy. Bryen et al. (2021) found a pathogenic deep intronic MTM1 variant that activates a pseudo-exon encoding a nonsense codon resulting in severe X-linked myotubular myopathy. Moreover, Niall P. Keegan summarized 58 DMD pseudoexons from published reports (Keegan, 2020).

In the current study, we initially found a novel *dysferlin* deletion in the index patient but no other potentially disease-causing variants. As *dysferlin* is a very large gene with a genomic size of approximately 233 kbp, it is difficult to perform traditional mutation screening (Blandin et al., 2012). Analysis is further complicated by the huge mutation spectrum of *dysferlin*, especially putative splicing and missense mutations. So far, more than 300 variants have been detected. Although previous study have identified single heterozygous Dysferlin mutations as pathogenic causes of Dysferlinopathies, the majority of *dysferlin* variants is required to be homozygous or potentially compound for functional alterations and the incidence for Dysferlinopathies (Nguyen et al., 2007).

There is a considerable difference in prognosis for the two myopathies presented in the current study, MMD and EOTD. MMD progresses slowly and generally does not affect longevity. The prognosis is worse with earlier onset. However, for neither disease, curative therapies are available (Balint et al., 2018; Kokubu et al., 2019) and traditional treatments are solely symptomatic. Recent research inferred that a drug inducing Dysferlin expression in myocytes could represent a targeted during clinical treatment for Dysferlinopathy patients (Kokubu et al., 2019), which also indicates that gene therapy could be an effective future clinical avenue for MMD patients.

In this study, we integrated genome sequencing (WES and WGS) and RNA-seq to solve a clinical puzzle. We discovered novel compound heterozygous mutation of *dysferlin* and the *TOR1A* ΔE mutation in the same family, causing two similar but distinct hereditary muscular disorders in siblings. Powerful genomic technologies are providing new insights into the genetics underlying Mendelian traits. Integrated genome and transcriptome sequencing is ideal for solving this type of clinical problems.

## Data Availability Statement

The raw sequence data reported in this article are deposited in the Genome Sequence Archive in National Genomics Data Center, China National Center for Bioinformation/Beijing Institute of Genomics, Chinese Academy of Sciences (https://bigd.big.ac.cn/) under accession number: HRA000876.

## Ethics Statement

The studies involving human participants were reviewed and approved by Ethics Committee of Wuhan Union Hospital in China. The patients/participants provided their written informed consent to participate in this study. Written informed consent was obtained from the individual(s) for the publication of any potentially identifiable images or data included in this article.

## Author Contributions

FZu and YL conceived and designed the study. FZu, FZa, LH, and YL performed the experiments and analyzed the data. FZu, FZa, and YL wrote the manuscript. All authors contributed to the article and approved the submitted version.

## Conflict of Interest

The authors declare that the research was conducted in the absence of any commercial or financial relationships that could be construed as a potential conflict of interest.

## Footnotes

1 http://www.1000genomes.org/

2 http://exac.broadinstitute.org

## Supplementary Material

Supplementary Figure 1 **(A)** Hematoxylin and eosin staining showed variation in muscle fiber size and slight degeneration (Bar, 50 μm). **(B)** Modified Gomori trichrome staining also showed slight degeneration of muscle fibers (Bar, 50 μm). **(C)** Neuron specific enolase staining showed deeply stained in atrophic and degenerative muscle fibers (Bar, 50 μm). **(D)** Reduced form of nicotinamide-adenine dinucleotide staining showed the uneven distribution of oxidase in a few muscle fibers (Bar, 100 μm). **(E)** Dystrophin-C, **(F)** Dystrophin-N and **(G)** Dystrophin-R showed normal staining of the corresponding marker. **(H)** sarcoglycan-α, **(I)** sarcoglycan-β, **(J)** sarcoglycan-γ, **(K)** sarcoglycan-δ showed normal staining of the corresponding marker.

Supplementary Figure 2 Gel electrophoresis of RT-PCR product from II1’s and health control’s peripheral blood mononuclear cells.

## References

[B1] Anczukow O. Buisson M. Leone M. Coutanson C. Lasset C. Calender A. (2012). BRCA2 deep intronic mutation causing activation of a cryptic exon: opening toward a new preventive therapeutic strategy. *Clin. Cancer Res.* 18 4903–4909. 10.1158/1078-0432.CCR-12-1100 22753590

[B2] Balint B. Mencacci N. E. Valente E. M. Pisani A. Rothwell J. Jankovic J. (2018). Dystonia. *Nat. Rev. Dis. Primers* 4:25. 10.1038/s41572-018-0023-6 30237473

[B3] Bashir R. Britton S. Strachan T. Keers S. Vafiadaki E. Lako M. (1998). A gene related to Caenorhabditis elegans spermatogenesis factor fer-1 is mutated in limb-girdle muscular dystrophy type 2B. *Nat. Genet.* 20 37–42. 10.1038/1689 9731527

[B4] Bashir R. Strachan T. Keers S. Stephenson A. Mahjneh I. Marconi G. (1994). A gene for autosomal recessive limb-girdle muscular dystrophy maps to chromosome 2p. *Hum. Mol. Genet.* 3 455–457. 10.1093/hmg/3.3.455 8012357

[B5] Blandin G. Beroud C. Labelle V. Nguyen K. Wein N. Hamroun D. (2012). UMD-DYSF, a novel locus specific database for the compilation and interactive analysis of mutations in the dysferlin gene. *Hum. Mutat.* 33 E2317–E2331. 10.1002/humu.22015 22213072

[B6] Bressman S. B. Raymond D. Fuchs T. Heiman G. A. Ozelius L. J. Saunders-Pullman R. (2009). Mutations in THAP1 (DYT6) in early-onset dystonia: a genetic screening study. *Lancet Neurol.* 8 441–446. 10.1016/S1474-4422(09)70081-X 19345147 PMC3712754

[B7] Bryen S. J. Oates E. C. Evesson F. J. Lu J. K. Waddell L. B. Joshi H. (2021). Pathogenic deep intronic MTM1 variant activates a pseudo-exon encoding a nonsense codon resulting in severe X-linked myotubular myopathy. *Eur. J. Hum. Genet.* 29 61–66. 10.1038/s41431-020-00715-7 32862205 PMC7852879

[B8] Chen R. Qiu Z. Wang J. Yao Y. Huang K. Zhu F. (2021). DES mutation associated with cardiac hypertrophy and alternating bundle branch block. *HeartRhythm Case Rep.* 7 16–20. 10.1016/j.hrcr.2020.10.003 33505848 PMC7813788

[B9] Cheng F. B. Feng J. C. Ma L. Y. Miao J. Ott T. Wan X. H. (2014). Combined occurrence of a novel TOR1A and a THAP1 mutation in primary dystonia. *Mov. Disord.* 29 1079–1083. 10.1002/mds.25921 24862462

[B10] Cooper D. N. Krawczak M. Polychronakos C. Tyler-Smith C. Kehrer-Sawatzki H. (2013). Where genotype is not predictive of phenotype: towards an understanding of the molecular basis of reduced penetrance in human inherited disease. *Hum. Genet.* 132 1077–1130. 23820649 10.1007/s00439-013-1331-2 PMC3778950

[B11] Cummings B. B. Marshall J. L. Tukiainen T. Lek M. Donkervoort S. Foley A. R. (2017). Improving genetic diagnosis in Mendelian disease with transcriptome sequencing. *Sci. Transl. Med.* 9:eaal5209. 10.1126/scitranslmed.aal5209 28424332 PMC5548421

[B12] Desmet F. O. Hamroun D. Lalande M. Collod-Béroud G. Claustres M. Béroud C. (2009). Human Splicing Finder: an online bioinformatics tool to predict splicing signals. *Nucleic Acids Res.* 37:e67. 10.1093/nar/gkp215 19339519 PMC2685110

[B13] Dominov J. A. Uyan O. McKenna-Yasek D. Nallamilli B. R. R. Kergourlay V. Bartoli M. (2019). Correction of pseudoexon splicing caused by a novel intronic dysferlin mutation. *Ann. Clin. Transl. Neurol.* 6 642–654. 10.1002/acn3.738 31019989 PMC6469257

[B14] Dominov J. A. Uyan O. Sapp P. C. McKenna-Yasek D. Nallamilli B. Hegde M. (2014). A novel dysferlin mutant pseudoexon bypassed with antisense oligonucleotides. *Ann. Clin. Transl. Neurol.* 1 703–720. 10.1002/acn3.96 25493284 PMC4241797

[B15] Eidelberg D. Moeller J. R. Antonini A. Kazumata K. Nakamura T. Dhawan V. (1998). Functional brain networks in DYT1 dystonia. *Ann. Neurol.* 44 303–312. 9749595 10.1002/ana.410440304

[B16] Exposito R. R. Gonzalez-Dominguez J. Tourino J. (2018). HSRA: hadoop-based spliced read aligner for RNA sequencing data. *PLoS One* 13:e0201483. 10.1371/journal.pone.0201483 30063721 PMC6067734

[B17] Fitzgerald J. Feist C. Dietz P. Moore S. J. Basel D. (2020). A deep intronic variant activates a pseudoexon in the MTM1 gene in a family with X-Linked myotubular myopathy. *Mol. Syndromol.* 11 264–270. 10.1159/000510286 33505229 PMC7802444

[B18] Ghilardi M. F. Carbon M. Silvestri G. Dhawan V. Tagliati M. Bressman S. (2010). Impaired sequence learning in carriers of the DYT1 dystonia mutation. *Ann. Neurol.* 54 102–109. 10.1002/ana.10610 12838525

[B19] Gill N. K. Ly C. Kim P. H. Saunders C. A. Fong L. G. Young S. G. (2019). DYT1 dystonia patient-derived fibroblasts have increased deformability and susceptibility to damage by mechanical forces. *Front. Cell Dev. Biol.* 7:103. 10.3389/fcell.2019.00103 31294022 PMC6606767

[B20] Gonorazky H. Liang M. Cummings B. Lek M. Micallef J. Hawkins C. (2016). RNAseq analysis for the diagnosis of muscular dystrophy. *Ann. Clin. Transl. Neurol.* 3 55–60. 10.1002/acn3.267 26783550 PMC4704476

[B21] Hsiao Y. H. Bahn J. H. Lin X. Chan T. M. Wang R. Xiao X. (2016). Alternative splicing modulated by genetic variants demonstrates accelerated evolution regulated by highly conserved proteins. *Genome Res.* 26 440–450. 10.1101/gr.193359.115 26888265 PMC4817768

[B22] Hu Y. Mohassel P. Donkervoort S. Yun P. Bolduc V. Ezzo D. (2019). Identification of a novel deep intronic mutation in CAPN3 presenting a promising target for therapeutic splice modulation. *J. Neuromuscul. Dis.* 6 475–483. 10.3233/JND-190414 31498126 PMC7522968

[B23] Kamm C. (2006). Early onset torsion dystonia (Oppenheim’s dystonia). *Orphanet J. Rare Dis.* 1:48. 10.1186/1750-1172-1-48 17129379 PMC1693547

[B24] Kamm C. Fischer H. Garavaglia B. Kullmann S. Sharma M. Schrader C. (2008). Susceptibility to DYT1 dystonia in european patients is modified by the D216H polymorphism. *Neurology* 70 2261–2262. 18519876 10.1212/01.wnl.0000313838.05734.8a

[B25] Keegan N. P. (2020). Pseudoexons of the DMD Gene. *J. Neuromuscul. Dis.* 7 77–95. 10.3233/JND-190431 32176650 PMC7175933

[B26] Kokubu Y. Nagino T. Sasa K. Oikawa T. Miyake K. Kume A. (2019). Phenotypic drug screening for dysferlinopathy using patient-derived induced pluripotent stem cells. *Stem Cells Transl. Med.* 8 1017–1029. 10.1002/sctm.18-0280 31250983 PMC6766604

[B27] Kukurba K. R. Montgomery S. B. (2015). RNA Sequencing and Analysis. *Cold Spring Harb. Protoc.* 2015 951–969. 10.1101/pdb.top084970 25870306 PMC4863231

[B28] Lee J. Jung J. H. Kim W. W. Hwang S. O. Park J. Y. Jeong J. Y. (2016). Ultrasound-Guided laser ablation using multidirectional-firing fiber for papillary thyroid carcinoma: an Ex vivo study with evaluation of tumor cell viability. *Photomed. Laser Surg.* 34 300–304. 10.1089/pho.2016.4088 27149406

[B29] Lv H. Qu Q. Liu H. Qian Q. Zheng X. Zhang Y. (2020). Clinical, neuroelectrophysiological and muscular pathological analysis of chronic progressive external ophthalmoplegia. *Exp. Ther. Med.* 20 1770–1774. 10.3892/etm.2020.8822 32742407 PMC7388235

[B30] Marco-Puche G. Lois S. Benitez J. Triviño J. C. (2019). RNA-Seq perspectives to improve clinical diagnosis. *Front. Genet.* 10:1152. 10.3389/fgene.2019.01152 31781178 PMC6861419

[B31] Matos L. Canals I. Dridi L. Choi Y. Prata M. J. Jordan P. (2014). Therapeutic strategies based on modified U1 snRNAs and chaperones for Sanfilippo C splicing mutations. *Orphanet J. Rare Dis.* 9:180. 10.1186/s13023-014-0180-y 25491247 PMC4279800

[B32] Miller J. M. Enemark E. J. (2016). Fundamental characteristics of AAA+ Protein family structure and function. *Archaea An Int. Microbiol. J.* 2016 1–12. 10.1155/2016/9294307 PMC5039278 27703410

[B33] Nakamura A. (2019). Mutation-Based therapeutic strategies for duchenne muscular dystrophy: from genetic diagnosis to therapy. *J. Pers. Med.* 9:16. 10.3390/jpm9010016 30836656 PMC6462977

[B34] Naro C. Jolly A. Di Persio S. Bielli P. Setterblad N. Alberdi A. J. (2017). An orchestrated intron retention program in meiosis controls timely usage of transcripts during germ cell differentiation. *Dev. Cell* 41 82–93.e4. 10.1016/j.devcel.2017.03.003 28366282 PMC5392497

[B35] Nguyen K. Bassez G. Krahn M. Bernard R. Laforêt P. Labelle V. (2007). Phenotypic study in 40 patients with dysferlin gene mutations: high frequency of atypical phenotypes. *Arch. Neurol.* 64 1176–1182. 10.1001/archneur.64.8.1176 17698709

[B36] O’Riordan S. Cockburn D. Barton D. Lynch T. Hutchinson M. (2002). Primary torsion dystonia due to the TOR1A GAG deletion in an Irish family. *Ir. J. Med. Sci.* 171 31–32. 11993591 10.1007/BF03168938

[B37] Oshlack A. Robinson M. D. Young M. D. (2010). From RNA-seq reads to differential expression results. *Genome Biol.* 11 220. 10.1186/gb-2010-11-12-220 21176179 PMC3046478

[B38] Ozelius L. J. Bressman S. B. (2011). Genetic and clinical features of primary torsion dystonia. *Neurobiol. Dis.* 42 127–135. 21168499 10.1016/j.nbd.2010.12.012 PMC3073739

[B39] Ozelius L. J. Hewett J. W. Page C. E. Bressman S. B. Kramer P. L. Shalish C. (1997). The early-onset torsion dystonia gene (DYT1) encodes an ATP-binding protein. *Nat. Genet.* 17 40–48. 10.1038/ng0997-40 9288096

[B40] Pertea M. Kim D. Pertea G. M. Leek J. T. Salzberg S. L. (2016). Transcript-level expression analysis of RNA-seq experiments with HISAT, StringTie and Ballgown. *Nat. Protoc.* 11 1650–1667. 10.1038/nprot.2016.095 27560171 PMC5032908

[B41] Pertea M. Pertea G. M. Antonescu C. M. Chang T. Mendell J. Salzberg S. (2015). StringTie enables improved reconstruction of a transcriptome from RNA-seq reads. *Nat. Biotechnol.* 33 290–295. 25690850 10.1038/nbt.3122 PMC4643835

[B42] Requena T. Gallego-Martinez A. Lopez-Escamez J. A. (2017). A pipeline combining multiple strategies for prioritizing heterozygous variants for the identification of candidate genes in exome datasets. *Hum. Genomics* 11:11. 10.1186/s40246-017-0107-5 28532469 PMC5441048

[B43] Romero N. E. Matson S. W. Sekelsky J. (2016). Biochemical Activities and Genetic Functions of the Drosophila melanogaster Fancm Helicase in DNA Repair. *Genetics* 204 531–541. 27466228 10.1534/genetics.116.192534 PMC5068844

[B44] Shigemizu D. Fujimoto A. Akiyama S. Abe T. Nakano K. Boroevich K. A. (2013). A practical method to detect SNVs and indels from whole genome and exome sequencing data. *Sci. Rep.* 3:2161. 10.1038/srep02161 PMC3703611 23831772

[B45] Sundaram C. Meena A. K. Uppin M. S. Govindaraj P. Vanniarajan A. Thangaraj K. (2011). Contribution of muscle biopsy and genetics to the diagnosis of chronic progressive external opthalmoplegia of mitochondrial origin. *J. Clin. Neurosci.* 18 535–538. 10.1016/j.jocn.2010.06.014 21277779

[B46] Tilgner H. Knowles D. G. Johnson R. Davis C. A. Chakrabortty S. Djebali S. (2012). Deep sequencing of subcellular RNA fractions shows splicing to be predominantly co-transcriptional in the human genome but inefficient for lncRNAs. *Genome Res.* 22 1616–1625. 10.1101/gr.134445.111 22955974 PMC3431479

[B47] Tsokos M. Linnoila R. I. Chandra R. S. Triche T. J. (1984). Neuron-specific enolase in the diagnosis of neuroblastoma and other small, round-cell tumors in children. *Hum. Pathol.* 15 575–584. 10.1016/s0046-8177(84)80012-x 6373565

[B48] Vaz-Drago R. Custodio N. Carmo-Fonseca M. (2017). Deep intronic mutations and human disease. *Hum. Genet.* 136 1093–1111. 10.1007/s00439-017-1809-4 28497172

[B49] Xiong J. Tu W. Yan Y. Xiao K. Yao Y. Li S. (2019). Identification of a novel NOG missense mutation in a Chinese family with symphalangism and tarsal coalitions. *Front. Genet.* 10:353. 10.3389/fgene.2019.00353 31105738 PMC6499182

[B50] Yang H. Wang K. (2015). Genomic variant annotation and prioritization with ANNOVAR and wANNOVAR. *Nat. Protoc.* 10:1556. 10.1038/nprot.2015.105 PMC4718734 26379229

[B51] Yang H. Robinson P. N. Wang K. (2015). Phenolyzer: phenotype-based prioritization of candidate genes for human diseases. *Nat. Methods* 12 841–843. 26192085 10.1038/nmeth.3484 PMC4718403

[B52] Yang J. F. Wu T. Li J. Y. Li Y. J. Zhang Y. L. Chan P. (2009). DYT1 mutations in early onset primary torsion dystonia and Parkinson disease patients in Chinese populations. *Neurosci. Lett.* 450 117–121. 10.1016/j.neulet.2008.10.111 19038309

