# Supplementary figures and images for: Integrated Genome and Transcriptome Sequencing to Solve a Neuromuscular Puzzle: Miyoshi Muscular Dystrophy and Early Onset Primary Dystonia in Siblings of the Same Family
Source: Front Genet. 2021 Jul 2;12:672906. doi: 10.3389/fgene.2021.672906 (PMC8283672; doi:10.3389/fgene.2021.672906)

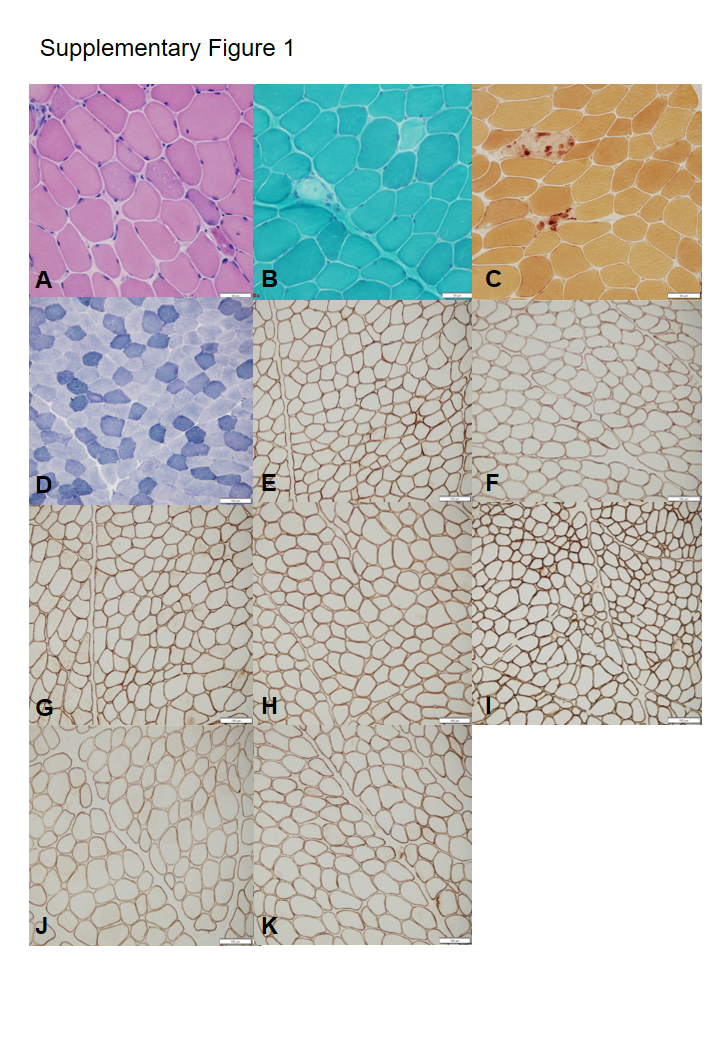

Supplement: Supplementary Figure 1 — (A) Hematoxylin and eosin staining showed variation in muscle fiber size and slight degeneration (Bar, 50 μm). (B) Modified Gomori trichrome staining also showed slight degeneration of muscle fibers (Bar, 50 μm). (C) Neuron specific enolase staining showed deeply stained in atrophic and degenerative muscle fibers (Bar, 50 μm). (D) Reduced form of nicotinamide-adenine dinucleotide staining showed the uneven distribution of oxidase in a few muscle fibers (Bar, 100 μm). (E) Dystrophin-C, (F) Dystrophin-N and (G) Dystrophin-R showed normal staining of the corresponding marker. (H) sarcoglycan-α, (I) sarcoglycan-β, (J) sarcoglycan-γ, (K) sarcoglycan-δ showed normal staining of the corresponding marker. [file Image_1.TIF]

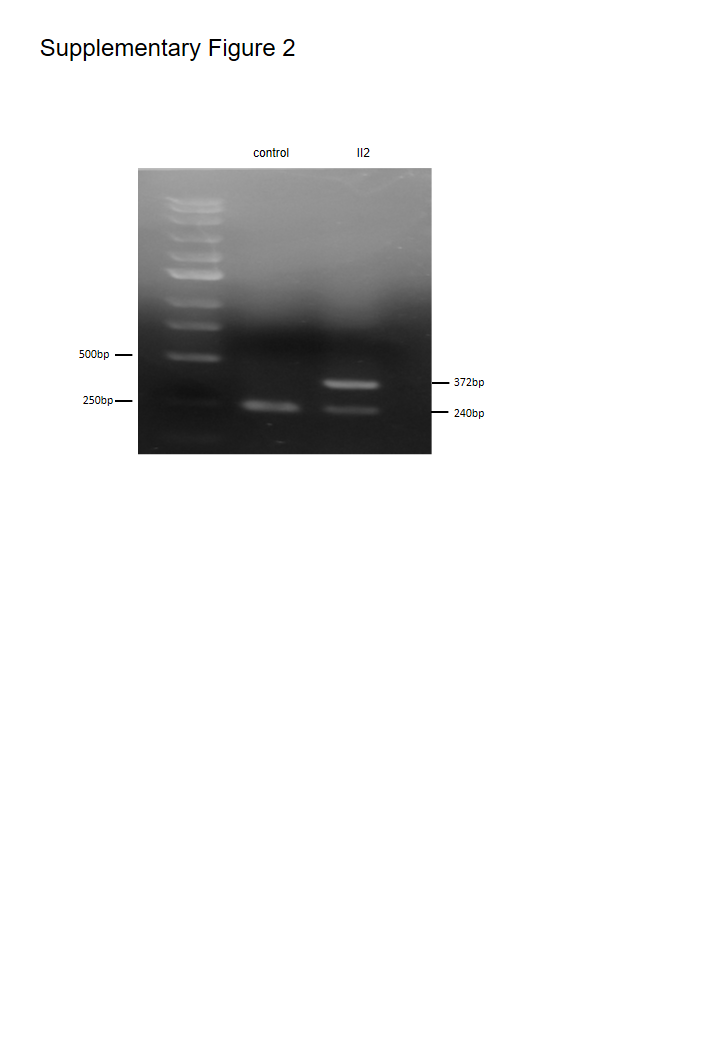

Supplement: Supplementary Figure 2 — Gel electrophoresis of RT-PCR product from II1’s and health control’s peripheral blood mononuclear cells. [file Image_2.TIF]
