# Supplementary material for: Integrated Genome and Transcriptome Sequencing to Solve a Neuromuscular Puzzle: Miyoshi Muscular Dystrophy and Early Onset Primary Dystonia in Siblings of the Same Family
Source: Front Genet. 2021 Jul 2;12:672906. doi: 10.3389/fgene.2021.672906 (PMC8283672; doi:10.3389/fgene.2021.672906)
Supplement: Supplementary file 3 [file Table_1.DOC]

**Supplemental Table 1 The number of variants after each prioritization step**

| **Step** | **II-1** | **II-2** |
| --- | --- | --- |
| Initial numbers of variants of WES data | 70,546 | 75,028 |
| 1. exclude variants outside exonic and splicing regions | 25,979 | 26,124 |
| 2. exclude synonymous variants | 13,859 | 13,704 |
| 3. exclude variants with MAF > 0.01 | 1,354 | 1,071 |
| 4. exclude non-conservative variants | 851 | 681 |
| 5. excluding benign or likely benign variants | 493 in 348 genes | 376 in 257 genes |
| 6. Phenolyzer (term ‘neuromuscular disease’) | DYSF: c.4404delC/p.I1469Sfs*17 | DYSF: c.4404delC/p.I1469Sfs*17  TOR1A:c.907_909del/p.303_303del |
